# 

**Published:** 1886-01

**Authors:** 


### OUR ADVERTISERS.

SURGICAL INSTRUMENTS.—C. W. Lane & Brother, 153 Broadway, New York, have an attractive advertisement in this issue of THE JOURNAL They will sell surgical instruments of all kinds for less than manufacturer's prices. See their advertisement and write for catalogue.

FAIRCHILD BROTHER & FOSTER, NEW YORK.—We take pleasure in calling attention to the advertisement of this firm, to be found next to "Society Reports," in this issue of THE JOURNAL. No firm in the country stands higher, and none deserves to. Their different pharmaceutical preparations are all of the very best, and physicians cannot go amiss in ordering such goods as they manufacture, by specifying Fairchild Bro. & Foster's.

NATROLITHIC WATER.—See advertisement of this justly popular mineral water in this issue of THE JOURNAL. It bears the endorsement of many of the most eminent physicians in this country. Send for samples of it.

THE place to buy pure drugs in Atlanta is at Charles O. Tyner's, corner Broad and Marietta streets, Bradfield & Ware's, 26 Whitehall, or Sharp & Bro., 202 Marietta street. They are all reliable houses, and any one ordering goods from them may feel assured that they will get just what they order, and at bottom figures.

SOMETHING OF INTEREST TO EVERY PHYSICIAN.—Tieman's surgical instruments can be bought in Atlanta at less than New York prices. Isaac Phillips, at No. 13 North Broad street, Atlanta, is the only house in the South dealing exclusively in surgical instruments. There is not a more responsible house in the South. See his advertisement elsewhere, which is full of interest to every physician, and write him for catalogue.

BUFFALO LITHIA WATER.—Of this well-known water Dr. Wm. H. Doughty, of Augusta, Ga., says: "I prescribe the Buffalo Lithia Water with the utmost confidence in all forms of indigestion, due to chronic catarrh of the mucous membrane, with excess of acid; also in the secondary or symptomatic dyspepsia of uterine and renal origin. It is the only reliable treatment known to me for the permanent relief of gravel and the antecedent conditions that determine it. In gout and rheumatism, I regard it as a remedy of great value. Over the nausea and vomiting of pregnancy, particularly in the latter months, where uræmic conditions are possibly established, and in puerperal convulsions, uræmia coexisting, it often exerts marked control. In hepatic disorders, especially where attended with jaundice or biliary calculi, it is an efficient remedy. In genito-urinary diseases, especially catarrh of the bladder in females, I have found it very efficacious. Whenever menstrual or uterine disorders are produced or intensified by such a state of the bladder, it becomes a valuable adjunct to other treatment. I have seen some very gratifying results from this water in chronic cutaneous diseases."

